# Elevated NS1 serum levels reduce CD119 expression and CXCL-10 synthesis in patients with dengue hemorrhagic fever

**DOI:** 10.1590/0037-8682-0577-2023

**Published:** 2024-07-29

**Authors:** Fernanda Gonçalves Garcia, Fernanda Rodrigues Helmo, Marcos Vinícius da Silva, Virmondes Rodrigues, Carlo José Freire Oliveira, Luciana de Almeida Silva Teixeira, Alexandre de Paula Rogério, David Nascimento Silva Teixeira

**Affiliations:** 1 Instituto de Ciências da Saúde, Laboratório de Pesquisa em Ativação Celular. Uberaba, MG, Brasil.; 2 Instituto de Ciências Biológicas e Naturais, Laboratório de Imunologia. Uberaba, MG, Brasil.; 3 Instituto de Ciências Biológicas e Naturais, Disciplina de Parasitologia. Uberaba, MG, Brasil.; 4 Instituto de Ciências da Saúde, Departamento de Clínica Médica. Uberaba, MG, Brasil.; 5 Instituto de Ciências Biológicas, Laboratório de Imunofarmacologia. Uberaba, MG, Brasil.

**Keywords:** Dengue, NS1 protein, CXCL-8, CXCL-10, IFN-γ

## Abstract

**Background:**

The intensity of *dengue virus* (DV) replication and circulating non-structural protein 1 (NS1) levels may promote changes in the human immune response and favor severe forms of infection. We investigated the correlations between NS1 with *CXCL-8*, *CXCL-10*, *IFN-γ*, and *IL-12p40* serum levels, and *IFN-γ* receptor α chain (CD119) expression, and CXCL10 production by peripheral blood mononuclear cells (PBMCs) stimulated with recombinant *IFN-γ* in DV-infected patients with different clinical forms.

**Methods:**

*Dengue virus* NS1, *CXCL-8*, *CXCL-10*, *IFN-γ*, and *IL-12p40* serum levels were measured in 152 DV-infected patients with different clinical forms and 20 non-infected individuals (NI) using enzyme-linked immunosorbent assay (ELISA). In addition, we investigated the *CXCL-10* production after *in vitro IFN-γ* stimulation of PBMCs from 48 DV-infected individuals (with different clinical forms of dengue fever) and 20 NI individuals using ELISA, and CD119 expression on CD14^+^ cells with flow cytometry.

**Results:**

Patients with dengue hemorrhagic fever (DHF) had significantly higher NS1, *CXCL-8*, and *CXCL-10* serum levels than those with classic dengue fever (DF). The response of PBMCs to *IFN-γ* stimulation was lower in patients with DHF than in those with DF or dengue with complications (DWC), with lower CD119 expression and reduced *CXCL-10* synthesis. In addition, these alterations are associated with high NS1 serum levels.

**Conclusions:**

Patients with DHF reported high NS1 levels, low CD119 expression, and low *CXCL-10* synthesis in PBMCs, which may be associated with infection progression and severity.

## INTRODUCTION

Dengue virus (DV), transmitted by the female *Aedes aegypti* mosquito, infects an estimated 100 to 400 million individuals worldwide annually^1^ ^,^ ^2^. It is endemic to Africa, the Americas, Southeast Asia, the Eastern Mediterranean, and the Western Pacific. In 2022, significant dengue fever outbreaks occurred globally, particularly in the Americas, where nearly three million cases were reported. Brazil has accounted for more than 2.3 million cases and reported 1,016 deaths^3^. 

Four serotypes of DV exist: DV-1, DV-2, DV-3, and DV-4^2^ ^,^ ^4^ ^,^ ^5^. It is a single-stranded RNA virus that synthesizes a polyprotein that produces three structural (C, prM, and E) and seven non-structural (NS) proteins (NS1, NS2a, NS2b, NS3, NS4a, NS4b, and NS5). The latter are involved in viral RNA replication^2^ ^,^ ^6^ ^,^ ^7^. NS1 is a viral glycoprotein occurring at different levels in the serum during the acute phase of infection and serves as a marker for early diagnosis of DF^8^ ^,^ ^9^. Furthermore, it could be associated with the etiopathogenesis of this disease^9^ ^,^ ^10^. 

The immune response to DV, although capable of containing infection and promoting recovery, may contribute to the development of more severe forms of the disease^10^ ^,^ ^11^ ^,^ ^12^. *IFN-γ* plays a central role in controlling viral replication and enhancing resistance to infection^13^ ^-^ ^19^ and the regulation of *IFN-γ* receptor expression on the cell surface could serve as a mechanism through which cells modify their response to *IFN-γ* ^19^ ^,^ ^20^. DV-infected individuals exhibit elevated *CXCL-10 (IP-10)* levels associated with the febrile period observed during the acute phase^21^ ^,^ ^22^ ^,^ ^23^; however, studies have not yet investigated the association between NS1 serum levels, inflammatory mediators, cellular response to *IFN-γ* and the development of different clinical forms of DF. Therefore, we hypothesized that NS1 levels could interfere with *IFN-γ*-induced *CXCL-10* synthesis and production of inflammatory mediators during an immune response to DV in humans. 

Elevated *CXCL-8 (IL-8)* levels have been linked to plasma leakage in patients with dengue hemorrhagic fever (DHF) and dengue shock syndrome (DSS). Individuals with thrombocytopenia and DV infections exhibit significantly higher *CXCL-8* levels^23^ ^-^ ^29^. One study using an *in vitro* model demonstrated that the synthesis of high levels of *CXCL-8* increased the risk of developing severe forms of the disease, particularly secondary infections^30^. However, the precise relationship between NS1 levels and changes in the innate immune response remains unclear. Thus, we assessed the circulating NS1 levels and their correlation with *CXCL-8*, *CXCL-10*, *IFN-γ*, and *IL-12p40* serum levels. In addition, *in vitro CXCL-10* production by peripheral blood mononuclear cells (PBMCs) in response to *IFN-γ* stimulation and *IFN-γ* receptor expression was evaluated in patients with different clinical forms of DF.

## METHODS

***Study group and case classification:*** A total of 213 patients were initially evaluated for participation by a team of infectious disease specialists at a dengue outpatient clinic. Of these, 51 were excluded from the study (24 did not have a positive laboratory diagnosis of DV infection, 1 had a positive serology for HIV, and 26 had incomplete material for analyses or inconclusive clinical and laboratory data). Therefore, the final group of patients diagnosed with DF comprised 152 patients (74 females and 78 males). Among them, 104 had samples collected only for serum, whereas the remaining 48 had samples collected for serum and PBMCs. Patients who sought care for suspected DV infection between the 2nd and 5th days of fever onset and who met the criteria were recruited into the study. Patients were followed and treated at the Dengue Outpatient Clinic of the Federal University of Triângulo Mineiro, Abadia Emergency Unit, and São Marcos Hospital between 2011 and 2014. Patients were classified after the analysis of all clinical data and laboratory results (obtained from at least two sample collections, one in the acute phase and the other in convalescence) by infectious disease specialists. A total of 106 DV-positive patients were classified as having classical DF, 31 as having dengue complications (DWC), and 15 as having DHF. Dengue cases were classified as DF or DHF according to the guidelines proposed by the World Health Organization (WHO)^31^. We applied the traditional classification because of the specific characteristics of our experimental work to test our hypothesis (evaluate whether elevated NS1 serum levels influence parameters specifically associated with the definition of DHF, such as platelet count, hematocrit, and others associated with immunopathogenesis). In addition, the 2009 WHO guidelines are more directly focused on clinical management and not broadly used in research^32^. For the control group, 20 healthy individuals (10 females and 10 males) without a current or recent history of fever or any other disease symptoms, who were not infected with DV, tested negative for NS1 and anti-DV IgM/IgG antibodies, and had not been vaccinated against yellow fever, were recruited and included as non-infected healthy controls (NI). Samples were obtained during the acute phase (days 2-5 after symptom onset) and at the beginning of the convalescent phase (days 9-12 after symptom onset). In addition, laboratory data (hemogram, NS1 levels, and anti-DV IgM/IgG), signs and symptoms (fever, headache, loop test, retro-orbital pain, diarrhea, rash, prostration, nausea, vomiting, epistaxis, gingivorrhagia, hematuria, petechiae, metrorrhagia, gastrointestinal bleeding, myalgia, arthralgia, severe abdominal pain, and painful hepatomegaly), and outcomes (hospitalization, death, and complications) were collected. Data on sex, age, and case classifications are presented in Table 1.

**TABLE 1:** Demographics and clinical data referring to *ex vivo* (NS1, *IFN-γ*, *IL-12p40*, *CXCL-8* and *CXCL-10* serum levels) and *in vitro* (CD119 expression and CXCL-10 production).

	DF	DWC	DHF	NI
***Ex vivo* evaluation**				
Age - x̅ (SD)	41.83 (21.73)	38.13 (17.83)	47.62 (21.29)	37.23 (14.85)
Male (*n*)	57	13	8	10
Female (*n*)	49	18	7	10
**Total**	**106**	**31**	**15**	**20**
Platelets x 10^3^/μL (SD)	169.31 (68.79)	99.34(49.51)	48.38(27.78)	253.2(64.7)
Hematocrit % (SD)	39.50 (4.53)	38.40 (8.27)	43.10 (5.57)	ND
IgM+ *n* (%)	83 (78.3)	25 (80.64)	11 (73.33)	0 (0)
***In vitro* evaluation**				
Age - x̅ (SD)	44.83 (20.12)	43.60 (18.04)	43.07 (21.67)	37.23 (14.85)
Male (*n*)	14	5	7	10
Female (*n*)	11	5	6	10
**Total**	**25**	**10**	**13**	**20**
Platelets x̅ 10^3^/μL (SD)	170.01 (57.63)	94.16(50.31)	42.33(24.32)	253.2(64.7)
Hematocrit % (SD)	40.20 (5.13)	39.21 (7.49)	42.90 (4.98)	ND
IgM+ *n* (%)	18 (72.0)	8 (80.0)	11 (73.33)	0 (0)

**DF:** dengue fever; **DWC:** dengue with complications; **DHF:** dengue hemorrhagic fever; **NI:** not infected; **x̅:** mean; **SD:** standard deviation; **ND:** not done.

***Blood samples collection and processing:*** During the acute phase, 5 mL of blood without anticoagulants was collected from 152 DV-infected individuals and 20 NI healthy controls to obtain serum samples. In addition, 20 mL of heparinized peripheral venous blood was collected during the acute phase from 48 DV-infected patients and 20 NI healthy individuals for PBMC isolation. During the convalescent phase, 5 mL of blood without anticoagulants was collected from 152 DV-infected individuals to obtain serum samples. All serum samples, collected without anticoagulant, were centrifuged at 500 ×g for 10 min at 21^o^C and stored in aliquots at −86°C until further use.

***Dengue fever serological diagnosis:*** Dengue fever-specific IgM and IgG were detected in serum samples using the Panbio^TM^ dengue-specific IgM and IgG capture enzyme-linked immunosorbent assay (ELISA) kit (Abbott Laboratories Inc., Abbott Park, IL, USA) according to the manufacturer’s instructions. The presence of DV NS1 antigen was determined using Platelia™ Dengue NS1 Ag immunoenzymatic assay (Bio-Rad Laboratories Inc., Marnes-la-Coquette, France) using the qualitative protocol performed according to the manufacturer’s instructions. 

***Serum soluble NS1 levels:*** NS1 serum levels were measured using a quantitative protocol of Platelia™ Dengue NS1 Ag enzyme immunoassay (Bio-Rad Laboratories, Marnes-la-Coquette, France) according to the manufacturer’s instructions. The quantitative protocol (Bio-Rad technical support version 05/2008 of Platelia Dengue NS1 Ag: quantitative detection of DV NS1 antigen in human serum or plasma by enzyme immunoassay) used a different conjugate dilution (1:5000) and a specific formula to calculate NS1 units based on human recombinant NS1 (positive control) and the calibrators of the kit. NS1 serum levels were calculated by multiplying the OD of the sample tested using the 1:5000 diluted conjugate by 150 as follows: Sample NS1 (BRU/mL) = Sample OD × 150/R4m, where R4m is the mean OD value obtained from cut-off control duplicates. This protocol was used for all samples confirmed positive using the qualitative protocol, and the results are expressed in Bio-Rad units per milliliter (BRU/mL).

***Isolation and culture of peripheral blood mononuclear cells (PBMCs):*** Peripheral blood (20 mL) was collected in heparinized tubes from 48 DV-positive patients and 20 NI control individuals. PBMCs were isolated using Ficoll-Hypaque density gradient centrifugation (Sigma-Aldrich) at 400 ×g for 30 min at 21°C. The cells were resuspended in the Roswell Park Memorial Institute (RPMI) 1640 medium (Lonza, Walkersville, MD, USA) supplemented with 50 mM HEPES buffer (Sigma-Aldrich), 10% inactivated fetal calf serum (Sigma-Aldrich), 1% (2 mM) L-glutamine (Sigma-Aldrich), and 100 U/mL penicillin/streptomycin (Sigma-Aldrich) to a final concentration of 1 × 10^6^ cells/mL. PBMCs were cultured in 24-well microplates (at 37°C/5% CO_2_) and stimulated with recombinant *IFN-γ* (R&D Systems, Inc., Minneapolis, MN, USA) at concentrations of 1, 5, and 10 ng/mL. After 18 h, cell culture supernatants were collected and stored in aliquots at −86°C for cytokine titration.

***Cell Viability Determination:*** The PBMC viability in culture and after *IFN-γ* stimulation was quantified by trypan blue exclusion test and their ability to reduce 3-(4,5-dimethylthiazol-2-yl)-2,5-diphenyl-tetrazolium bromide (MTT: 5 mg/mL, Sigma-Aldrich) to formazan for 4 h at 37^o^C/5% CO_2_. The MTT formazan dye was dissolved by incubation in DMSO (Merck, Berlin, Germany), and its concentration was determined spectrophotometrically at 570 nm.

***Cytokine and chemokine quantification:*** *IFN-γ*, *IL-12p40*, *CXCL-8*, and *CXCL-10* concentrations in serum and 18 h cell culture supernatants were measured using ELISA kits with monoclonal antibody pairs according to the manufacturer’s specifications (BD Biosciences, San Jose, CA, USA). The absorbance was determined using a Modulus microplate reader (Promega, Madison, WI, USA), and the absorbance at 450 nm was subtracted from that at 570 nm. Cytokine/chemokine concentrations were calculated and the results are expressed in pg/mL.

***Analysis of IFN-γR1 receptor expression: IFN-γR1*** receptor profiles were analyzed by incubating PBMCs (5 × 10^5^ cells/mL) in a staining buffer (Dulbecco’s phosphate-buffered saline supplemented with 1% inactivated fetal calf serum, Sigma-Aldrich) together with monoclonal antibodies (BD Biosciences) against CD14 and CD119 (*IFN-γR1*) at 4^o^C for 30 min. Next, cells were washed twice (300 × g for 5 min at 4°C) and resuspended in staining buffer (500 μL). An Accuri cytometer (Becton Dickinson and Company, Franklin Lakes, NJ, USA) was used for event acquisition, and the data were analyzed using FlowJo (Tree Star Inc., Ashland, OR, USA). 

***Cytometry analysis strategy:*** The CD119 (*IFN-γR1*) expression on CD14^+^ PBMCs was obtained by drawing a gate on FL1 (CD14^+^ FITC fluorescence) versus the SSC parameter. The CD14^+^ cells in region 1 (R1) were further analyzed in a PE fluorescence channel (FL2) representing CD119 expression.

***Statistical analyses:*** Data were analyzed using GraphPad Prism 7.0 (GraphPad Software, La Jolla, CA, USA). The Mann-Whitney *U* test was used for comparisons between two groups, and the Kruskal-Wallis test was used for comparisons between three or more groups. Spearman’s correlation coefficient (*r*) was calculated to determine the linear association between the two variables. The significance level was set at 5%. 

***Ethics statement:*** The study protocol was approved by the Ethics Committee of the Federal University of Triângulo Mineiro, Uberaba, Minas Gerais State, Brazil (protocol n^o^. 851). All participants provided written informed consent.

## RESULTS

### ● Circulating NS1 levels during the acute phase and convalescence

**FIGURE 1:**
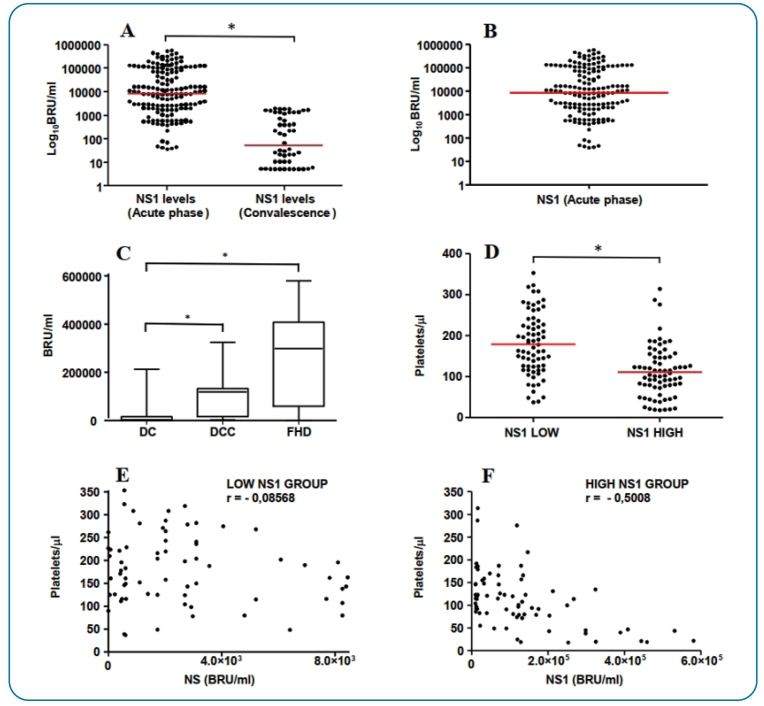
Serum concentration distribution of dengue virus non-structural protein 1 (NS1) and platelet number in infected individuals (*n* = 152). **(A)** NS1 serum levels in acute febrile phase (days 2 to 4) and beginning of convalescence (day 9). **(B)** NS1 circulating serum levels during the acute phase, where the line represents the median of 152 DV-infected individuals. **(C)** NS1 serum levels in the acute febrile phase in individuals with dengue fever (DF), dengue with complications (DWC), and dengue hemorrhagic fever (DHF) in BRU/mL. **(D)** Platelet count/µL in infected individuals with low (NS1 < 8.4 × 10^3^ BRU/mL) and high NS1 serum levels (NS1 ≥ 8.4 × 10^3^ BRU/mL) during the acute phase. **(E)** Spearman’s correlation between NS1 levels and platelet count/µL in infected individuals from the low NS1 group during the acute phase. **(F)** Spearman’s correlation between NS1 levels and platelet count/µL in infected individuals from the high NS1 group during the acute phase. The (*) symbol between the two groups indicates a statistically significant difference (*p* < 0.05).

The NS1 antigen was detected in the circulation of DV-positive patients from the acute phase to the recovery period, with significantly higher viral protein levels observed in the acute phase (*p-value* < 0.01) than in the initial convalescent period after infection (Figure 1A). During the acute phase, NS1 serum levels varied significantly among individuals, ranging from 0.2 × 10^1^ to 6.0 × 10^5^ BRU/mL (Figure 1B). Patients with DHF and DWC exhibited higher NS1 levels than those with DF (*p-value* < 0.01) (Figure 1C). 

The NS1 serum levels showed a heterogeneous distribution among DV-positive patients (median = 8.4 × 10^3^ BRU/mL) during the acute phase (Figure 1B), and two groups were established: patients with low NS1 serum levels (NS1 levels < median) and those with high NS1 serum levels (NS1 levels ≥ median). Individuals with high NS1 serum levels had lower platelet count/μL (*p-value* < 0.01) than those with reduced NS1 serum levels (Figure 1D). A negative correlation was observed during the acute phase (Figure 1E and 1F) between platelet counts and circulating NS1 levels, especially in the high NS1 patient group (Figure 1F, *r* = −0.5008).

### ● *IFN-γ, IL-12p40, CXCL-8,* and *CXCL-10* serum levels during the acute phase and convalescence

**FIGURE 2:**
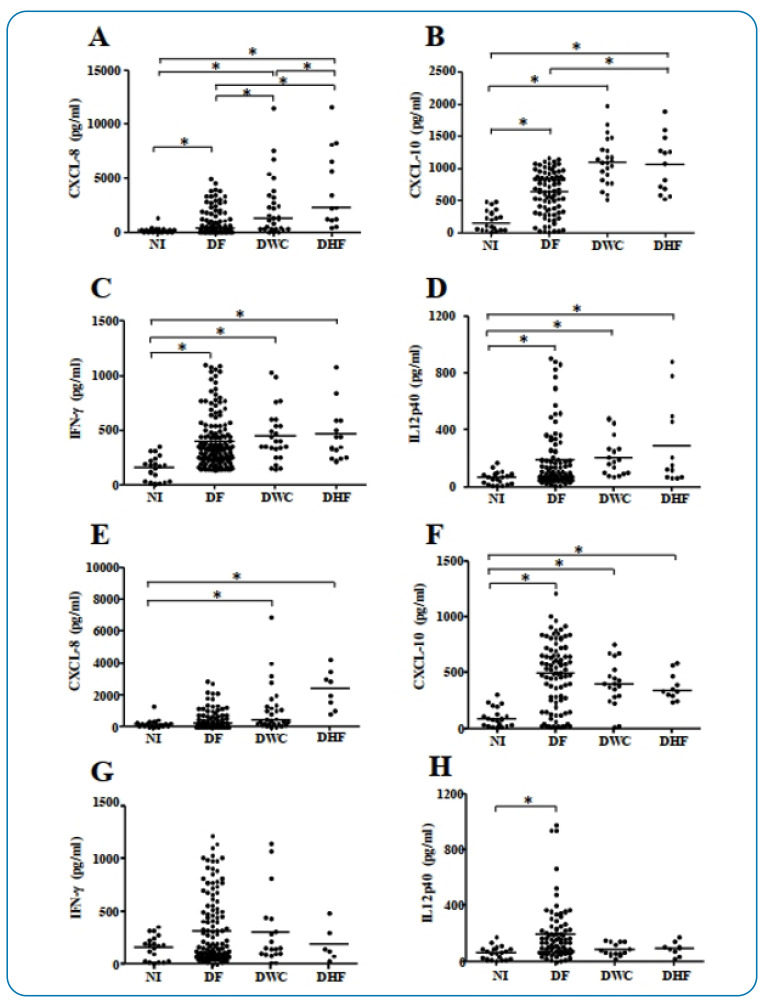
CXCL-8, CXCL-10, IFN-γ, and IL-12p40 serum concentrations during the acute phase **(A to D)** and convalescence **(E to H)** in DV-infected individuals (*n* = 152). **(A, E)** CXCL-8, **(B, F)** CXCL-10, **(C, G)** IFN-γ, and **(D, H)** IL-12p40 serum levels (pg/mL) in patients with dengue fever (DF), dengue with complications (DWC), and dengue hemorrhagic fever (DHF), and in non-infected individuals (NI, *n* = 20). The (*) symbol between the two groups indicates a statistically significant difference (*p* < 0.05).

During the acute febrile phase of infection, patients with DHF and DWC exhibited elevated *CXCL-8* (*p-value* < 0.01) compared with those with DF (Figure 2A). Furthermore, patients with DHF and DWC exhibited increased CXCL-10 serum levels (*p-value* < 0.01) compared to those with DF (Figure 2B). Notably, *CXCL-8*, *CXCL-10*, *IFN-γ,* and *IL-12p40* serum levels in DHF, DWC, and DF were higher than those in NI individuals (Figure 2A-D). 

At the beginning of the convalescence period (day 9), as shown in Figure 2 E , patients with DHF exhibited a sustained elevation in *CXCL-8* levels compared to DF and NI individuals (*p-value* < 0.01). Similarly, *CXCL-10* levels were higher in patients with DHF than in NI individuals (Figure 2F, *p-value* < 0.01). In patients with DWC, both *CXCL-8* (*p-value* < 0.01) and *CXCL-10* (*p-value* < 0.01) serum levels remained elevated compared with those in NI individuals (Figure 2E and F). In the DF group, *CXCL-10* and *IL-12p40* levels were elevated (*p-value* < 0.01) compared with those in the NI group (Figure 2E, F and H). In contrast, *IFN-γ* serum levels did not differ significantly (*p-value* = 0.52) among the groups (Figure 2G).

### ● Serum *IFN-γ*, *IL-12p40*, *CXCL-8*, and *CXCL-10* levels in relation to NS1 levels

**FIGURE 3:**
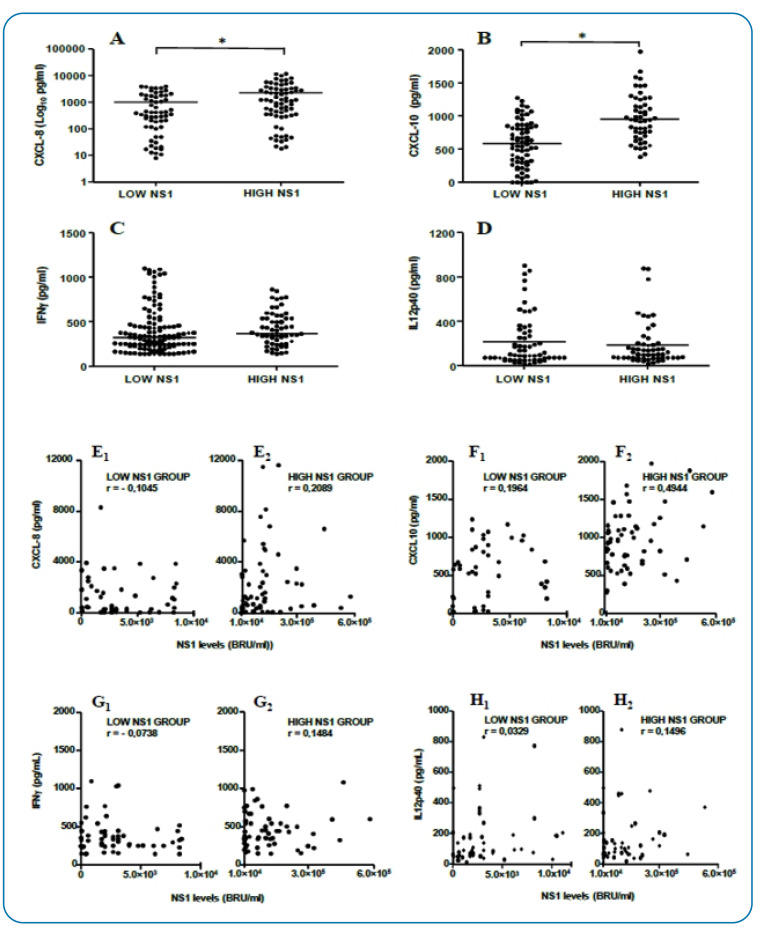
CXCL-8 **(A)**, CXCL-10 **(B)**, IFN-γ **(C)**, and IL-12p40 (D) serum concentrations (pg/mL) in individuals with low NS1 (< 8.4 × 10^3^ BRU/mL) or high NS1 serum levels (≥ 8.4 × 10^3^ BRU/mL) during the acute phase of the infection. Spearman’s correlations between CXCL-8 **(E**_1_ **)**, CXCL-10 **(F**_1_ **)**, IFN-γ **(G**_1_ **)**, and IL-12p40 **(H**_1_ **)** concentrations versus NS1 serum levels in dengue virus-infected individuals from the low NS1 group during the acute phase and Spearman’s correlations between CXCL-8 **(E**_2_ **)**, CXCL-10 **(F**_2_ **)**, IFN-γ **(G**_2_ **)**, and IL-12p40 **(H**_2_ **)** concentrations versus NS1 serum levels in dengue virus-infected individuals from the high NS1 group during the acute phase (*n* = 152). The (*) symbol between the two groups indicates a statistically significant difference (*p* < 0.05).

*IFN-γ*, *IL-12p40*, *CXCL-8*, and *CXCL-10* serum levels were evaluated according to NS1 levels *in vivo* during the acute phase of DV infection. Patients with high NS1 levels exhibited higher concentrations of *CXCL-8* (*p-value* < 0.01) and *CXCL-10* (*p-value* < 0.01) than those with low NS1 levels (Figure 3A and B). These findings were supported by positive correlations observed in high NS1 patient groups between *CXCL-8* (*r* = 0.2089) and *CXCL-10* (*r* = 0.4944) with NS1 serum levels in DV-positive patients (Figure 3E_2_ and 3F_2_).

However, no significant differences were observed in *IFN-γ* (*p-value* = 0.51) and *IL-12p40* (*p-value* = 0.95) serum levels concerning NS1 concentration (Figure 3C and D). Similarly, weak positive correlations were observed between *IFN-γ* (*r* = 0.1484), *IL-12p40* (*r* = 0.1496), and NS1 levels in the high NS1 patient group (Figure 3G_2_ and 3H_2_). 

### ● Induction of *CXCL-10* synthesis by stimulation of the *IFN-γ* receptor with recombinant *IFN-γ*

**FIGURE 4:**
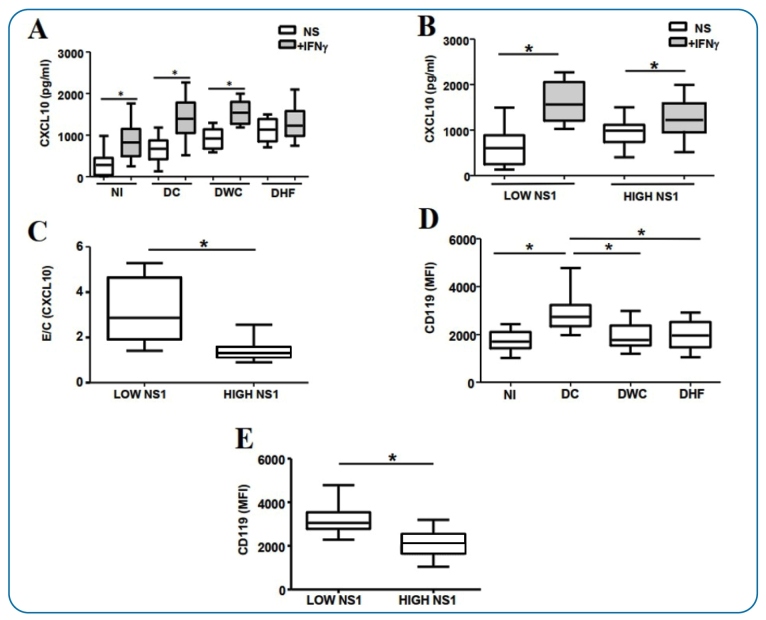
*CXCL-10* synthesis after recombinant *IFN-γ* stimulation and *IFN-γ* receptor alpha chain (*IFN-γRα* or CD119) expression in peripheral blood mononuclear cells (PBMCs) from infected individuals (*n* = 48). **(A)** *CXCL-10* synthesis (pg/mL) in individuals with dengue fever (DF), dengue with complications (DWC), and dengue hemorrhagic fever (DHF), and in 18 non-infected individuals (NI) with and without recombinant *IFN-γ* stimulation. **(B)** *CXCL-10* synthesis (pg/mL) in individuals with low (NS1 < 8.4 × 10^3^ BRU/mL) and high NS1 serum levels (NS1 ≥ 8.4 × 10^3^ BRU/mL) with and without *in vitro* recombinant *IFN-γ* stimulation. **(C)** Cellular activation **(E/C)**, where E stands for *CXCL-10* synthesis after recombinant *IFN-γ* stimulation, and C stands for *CXCL-10* synthesis without recombinant *IFN-γ* stimulation. **(D)** CD119 (*IFN-γRα*) expression per CD14^+^ monocytes in patients with dengue fever (DF), dengue with complications (DWC), and dengue hemorrhagic fever (DHF), and in not infected (NI) individuals. **(E)** CD119 (*IFN-γRα*) expression per CD14^+^ monocytes in individuals with low (NS1 < 8.4 × 10^3^ BRU/mL) and high NS1 serum levels (NS1 ≥ 8.4 × 10^3^ BRU/mL) in the acute phase. The * symbol between the two groups indicates a statistically significant difference (*p* < 0.05).

The *CXCL-10* synthesis by PBMCs after stimulation with recombinant *IFN-γ* was investigated in DV-infected and NI individuals during the acute phase (Figure 4A). Cells from the DF, DWC, and NI groups stimulated with *IFN-γ* (+) exhibited higher *CXCL-10* production than non-stimulated cells (DF, *p-value* < 0.01; DWC, *p-value* < 0.01; NI, *p-value* < 0.01). The DHF, DWC, and DF *IFN-γ* (+) groups demonstrated higher *CXCL-10* synthesis than the NI *IFN-γ* (+) group (*p-value* < 0.01). Similar results were observed in the DHF and DWC non-stimulated *IFN-γ* (−) groups compared to the NI *IFN-γ* (−) group (*p-value* < 0.01). Although cells from DHF patients displayed a higher baseline *CXCL-10* synthesis (Figure 4A), no significant increase in *CXCL-10* production was detected after stimulation with recombinant *IFN-γ* (*p-value* = 0.57)

Patients with low and high NS1 serum levels stimulated with *IFN-γ* (Figure 4B) exhibited higher *CXCL-10* synthesis than non-stimulated cells (low NS1, *p-value* < 0.01; high NS1, *p-value* < 0.01). Individuals with increased NS1 serum levels displayed a lower cellular response (lower increase in *CXCL-10* synthesis) after stimulation with *IFN-γ* when compared to those with lower circulating NS1 concentrations (Figure 4C). These results indicate a significantly lower cell response to *IFN-γ* (*p-value* < 0.01) in the group with increased NS1 levels, as evidenced by the lower E/C *CXCL-10* ratio [*IFN-γ* (+)/*IFN-γ* (−)].

### ● *IFN-γ* receptor alpha chain expression in CD14 ^+^ **cells**

We investigated whether the reduced cellular response to *IFN-γ* in DHF was associated with a modulation of the *IFN-γ* receptor α chain expression (CD119 or *IFN-γR1*) in CD14^+^ cells (Figure 4D and 4E).

As shown in Figure 4D, during the acute phase, CD14^+^ cells in patients with DF exhibited higher CD119 expression than those in patients with DHF (*p-value* < 0.01), DWC (*p-value* < 0.01), or NI individuals (*p-value* < 0.01). CD14^+^ cells from patients with higher NS1 serum levels exhibited lower CD119 expression (*p-value* < 0.01) than those from patients with lower NS1 concentrations (Figure 4E).

## DISCUSSION

NS1 serum concentrations displayed significant heterogeneity during the acute phase of DV infection, with patients having higher NS1 levels than those with DF. Thus, individuals with DF may have a more effective immune response during the acute phase, facilitating DV clearance and the subsequent reduction in soluble viral antigens throughout infection. High NS1 serum levels may contribute to the development of severe hemorrhagic forms such as DHF because NS1 may induce abundant anti-NS1 antibodies that cross-react with platelets, causing thrombocytopenia and altered coagulation^8^ ^-^ ^10^ ^,^ ^12^ ^,^ ^33^. Within this context, our findings are consistent with those of previous studies in which individuals with DHF exhibited elevated NS1 serum levels compared to those with DF^9^ ^,^ ^10^ ^,^ ^12^ ^,^ ^34^. The synthesis of anti-NS1 antibodies can lead to cross-reactivity against epitopes present in platelets and vascular endothelium, inducing apoptosis of endothelial cells, platelet aggregation, and complement system-mediated lysis. In addition, these processes could contribute to hemorrhage and plasma leakage during DV infection^35^ ^-^ ^37^. 

We observed that infected individuals with increased NS1 levels exhibited reduced platelet counts, as reported previously^33^ ^-^ ^34^. The literature reports that anti-DV NS1 antibodies bind to protein disulfide isomerase on the platelet surface to inactivate this protein and αIIbβ2 integrin, which inhibits platelet adhesion and aggregation^35^ ^-^ ^38^. This process also promotes increased phagocytosis due to the opsonization of platelets^39^. Although endothelial dysfunction is complex and poorly understood, our findings suggest that high NS1 serum levels detected during the acute phase of infection could be associated with the development of severe forms, such as DHF/DSS. Our study demonstrated that patients who developed severe forms, such as DHF, had extremely high serum levels of NS1 during the acute phase. High concentrations of NS1 can produce abundant anti-NS1 antibodies that can cross-react with epitopes present in endothelial cells, causing temporary dysfunction of the endothelium^35^ ^-^ ^39^ Furthermore, it can bind to platelets, causing numerous alterations such as opsonization, agglutination, and microthrombi formation^37^ ^-^ ^39^. Thus, these factors may be associated with the development of severe forms, such as DHF/DSS, during the beginning of the defervescence phase of DV infection. However, further studies are required to describe the mechanisms involved in detail and reinforce these hypotheses.

Serum NS1 levels varied significantly from patient to patient over a very wide range (from 0.22 × 10^1^ to 6 × 10^5^ BRU/mL). Thus, we investigated whether the different levels of serum NS1 were correlated with changes in the production of important host immune mediators during DV infection. We evaluated four key molecules (CXCL-8, CXCL-10, *IFN-γ* and IL12p40). CXCL-8 chemokine was selected largely because of its vasoactive properties and the possibility of its association with the development of severe forms of infection^23^ ^-^ ^30^. CXCL-10 was selected as a direct marker of cellular response to *IFN-γ* because CXCL-10, also known as induced protein 10 (IP-10), is induced directly in response to *IFN-γ* action^21^ ^,^ ^22^ ^,^ ^23^. The choice of IL12 and *IFN-γ* dependent on their fundamental participation in efficient antiviral responses^13^ ^-^ ^19^. Compared to NI individuals, DV-infected individuals exhibited elevated *CXCL-8*, *CXCL-1*0, *IFN-γ*, and *IL-12p40* serum levels during the acute phase. Patients with DHF and DWC exhibited higher *CXCL-8* and *CXCL-10* levels than those with DF. During convalescence, individuals with DHF and DWC maintained elevated *CXCL-8* and *CXCL-10* levels, whereas those with DF showed sustained *CXCL-10* and *IL-12p40* levels. *In vivo* and *in vitro* studies have demonstrated increased serum *CXCL-8* ^25^ ^-^ ^28^ ^,^ ^30^ ^,^ ^40^ and *CXCL-10* ^22^ ^,^ ^23^ ^,^ ^40^ ^,^ ^41^ levels during DV infection, both during the acute phase and defervescence, particularly in the more severe forms of the disease^30^ ^,^ ^40^ ^,^ ^42^ ^,^ ^43^ ^,^ ^44^. 

Studies have highlighted increased *IFN-γ* and *IL-12* levels in DV infections across different clinical forms^15^ ^,^ ^16^ ^,^ ^45^ ^,^ ^46^. Individuals with DF maintain elevated serum *IFN-γ* and *IL-12* levels, which promote infection^15^ ^,^ ^16^ ^,^ ^17^ ^,^ ^45^ ^,^ ^46^. However, individuals with DHF experience peaks of *IFN-γ* (mainly before plasma leakage)^17^ and *IL-12* ^46^, followed by reduced serum levels. Individuals with high NS1 levels exhibited elevated *CXCL-8* and *CXCL-10* levels, but not *IFN-γ* and *IL-12p40* levels. The concentrations of both chemokines increased proportionally with the NS1 levels, indicating that NS1 may affect certain parameters of the antiviral response during acute infection. Increases in *CXCL-8* synthesis, vascular permeability, and plasma leakage are possibly triggered by systemic alterations^40^ ^,^ ^47^, and anti-DV NS1 antibodies promote *CXCL-8* synthesis after endothelial activation^48^. Therefore, high levels of *CXCL-10*, *CXCL-8,* and NS1 may be associated with excessive and unregulated inflammatory responses during DV infection.

We demonstrated that PBMCs from individuals with DHF exhibited a diminished response to *IFN-γ*, as indicated by a decreased *CXCL-10* synthesis compared to individuals with DF and DWC. Similarly, individuals with high NS1 levels exhibited a poor cellular response to *IFN-γ*, resulting in no increase in *CXCL-10* synthesis after *in vitro* stimulation. In contrast, patients with reduced NS1 levels demonstrated increased *IFN-γ* response. We demonstrated that DV suppresses cellular immunity during the peak viral replication by temporarily reducing *IFN-γ* synthesis by PBMCs, probably affecting the stability and activity of the *IFN-γ/IFN-γ* receptor system^18^. Dendritic cells (DC) infected *in vitro* with DV produce less *CXCL10* than non-infected DCs, and the DC maturational state is modified by the presence of DV^49^. This affects the etiopathogenesis of the disease because the reduced capacity of DV-infected cells to stimulate CD4^+^ T lymphocytes impacts the efficiency of the immune response, causing reduced control of the viral load^49^. The evasion mechanisms employed by DV may affect the IFN-mediated antiviral response. Specifically, they hinder the phosphorylation of Tyk2 tyrosine kinase (a STAT activating molecule)^50^, and the viral protease NS2B3 acts by reducing *IFN-γ* synthesis^51^. 

Therefore, the observed lower response to the *IFN-γ* stimulus in PBMCs from individuals with DHF suggests a modulation of the *IFN-γ/IFN-γ* receptor system, possibly associated with high NS1 concentrations, impairment of viral replication control mechanisms, and modulation of inflammatory response pathways. Our findings support the hypothesis that changes induced by high levels of NS1 affect the *IFN-γ* signaling pathway, and unregulated signaling may also lead to severe inflammation^52^. 

In addition to increased NS1 serum levels and a reduced cellular response to *IFN-γ* detected in DHF patients, reduced *IFN-γ* receptor CD119 expression was observed in CD14^+^ cells in these patients. This reduction was associated with a higher concentration of circulating NS1 because of a lower expression of CD119 in CD14^+^ monocytes in the high NS1 patient group. Although the involvement of NS1 in viral RNA replication has been recognized, its precise function in DF pathogenesis remains incompletely elusive^8^ ^,^ ^53^ ^,^ ^54^ ^,^ ^55^. Further investigations are required to explore the potential mechanisms by which CXCL-8, a powerful vasoactive chemokine, alters the immune response. Moreover, increased NO levels produced during the inflammatory response may interfere with CD119 expression in infected cells^56^. Our results suggest a DV escape mechanism subverts the host immune response, particularly the cellular response to *IFN-γ* stimulation and *CXCL-10* synthesis.

Among the limitations of our study is the non-detection of the predominant virus serotype, although the identification of specific circulating DV serotypes was not the main concern of our study. During this epidemic, official agencies from our state and municipality analyzed only a small percentage of samples from our city, and DV serotype 1 was the most frequently found in our region. We simultaneously serotyped a few of the 152 samples included in the study (*n* = 8) and detected serotype 1 in all analyzed patients. In addition, the panel of cytokines and chemokines investigated could have been broader. However, considering numerous biological samples analyzed in this study, we focused on key mediators associated with NS1 levels, course of infection, and development of DHF. In contrast, our work opens perspectives for investigating the molecular mechanisms involved in the likely suppression of the cellular response to *IFN-γ* by NS1 during the critical period of the acute phase and defervescence. Another open perspective is to investigate the possible function of anti-NS1 antibodies in the immunopathogenesis of dengue.

In conclusion, individuals with severe forms of DF exhibit immune response alterations during the acute phase, including increased serum NS1 levels that are associated with platelet reduction, higher concentrations of *CXCL-8* and *CXCL-10*, and reduced *IFN-γ* levels. Lower expression of CD119 in CD14^+^ monocytes detected in DHF patients when compared to DF patients, is related to a diminished cellular response to *IFN-γ* causing reduced *CXCL-10* synthesis. Therefore, the combination of alterations detected may contribute to the progression and severity of DF. 

## References

[1] WHO (2023). Dengue and severe dengue.

[2] Guzman MG, Harris E (2015). Dengue. Lancet.

[3] PAHO (2023). Epidemiological Update Dengue, chikungunya and Zika Brasília: Pan
American Health Organization.

[4] Rico-Hesse R (1990). Molecular evolution and distribution of dengue viruses type 1 and
2 in nature. Virology.

[5] Holmes EC, Twiddy SS (2003). The origin, emergence and evolutionary genetics of dengue
virus. Infect Genet Evol.

[6] Chambers TJ, Hahn CS, Galler R, Rice CM (1990). Flavivirus genome organization, expression, and
replication. Annual review of microbiology.

[7] Navarro-Sánchez E, Desprès P, Cedillo-Barrón L (2005). Innate immune responses to dengue virus. Arch Med Res.

[8] Muller DA, Young PR (2013). The flavivirus NS1 protein: molecular and structural biology,
immunology, role in pathogenesis and application as a diagnostic
biomarker. Antiviral Res.

[9] Carvalho DM, Garcia FG, Terra AP, Lopes Tosta AC, Silva Lde A, Castellano LR, Silva-Teixeira DN (2014). Elevated dengue virus nonstructural protein 1 serum levels and
altered toll-like receptor 4 expression, nitric oxide, and tumor necrosis
factor alpha production in dengue hemorrhagic Fever patients. J Trop Med.

[10] Perera DR, Ranadeva ND, Sirisena K, Wijesinghe KJ (2024). Roles of NS1 Protein in Flavivirus Pathogenesis. ACS Infect Dis.

[11] Espada-Murao LA, Morita K (2011). Dengue and soluble mediators of the innate immune
system. Tropical Medicine and Health.

[12] Ghetia C, Bhatt P, Mukhopadhyay C (2022). Association of dengue virus non-structural-1 protein with disease
severity: a brief review. Trans R Soc Trop Med Hyg.

[13] Kurane I, Innis BL, Nimmannitya S, Nisalak A, Meager A, Janus J, Ennis FA (1991). Activation of T lymphocytes in dengue virus infections. High
levels of soluble interleukin 2 receptor, soluble CD4, soluble CD8,
interleukin 2, and interferon-gamma in sera of children with
dengue. J Clin Invest.

[14] Green S, Pichyangkul S, Vaughn DW, Kalayanarooj S, Nimmannitya S, Nisalak A (1999). Early CD69 expression on peripheral blood lymphocytes from
children with dengue hemorrhagic fever. J. Infect. Dis.

[15] Chen LC, Lei HY, Liu CC, Shiesh SC, Chen SH, Liu HS (2006). Correlation of serum levels of macrophage migration inhibitory
factor with disease severity and clinical outcome in dengue
patients. Am J Trop Med Hyg.

[16] Restrepo BN, Isaza DM, Salazar CL, Ramírez R, Ospina M, Alvarez LG (2008). Serum levels of interleukin-6, tumor necrosis factor-alpha and
interferon-gamma in infants with and without dengue. Rev Soc Bras Med Trop.

[17] Priyadarshini D, Gadia RR, Tripathy A, Gurukumar KR, Bhagat A, Patwardhan S (2010). Clinical findings and pro-inflammatory cytokines in dengue
patients in Western India: a facility-based study. PloS one.

[18] Gunther VJ, Putnak R, Eckels KH, Mammen MP, Scherer JM, Lyons A (2011). A human challenge model for dengue infection reveals a possible
protective role for sustained interferon gamma levels during the acute phase
of illness. Vaccine.

[19] Novelli F, Bernabei P, Ozmen L, Rigamonti L, Allione A, Pestka S (1996). Switching on of the proliferation or apoptosis of activated human
T lymphocytes by IFN-gamma is correlated with the differential expression of
the alpha- and beta-chains of its receptor. J Immunol.

[20] Tau G, Rothman P (1999). Biologic functions of the IFN-gamma receptors. Allergy.

[21] Dejnirattisai W, Duangchinda T, Lin CL, Vasanawathana S, Jones M, Jacobs M (2008). A complex interplay among virus, dendritic cells, T cells, and
cytokines in dengue virus infections. J Immunol.

[22] de-Oliveira-Pinto LM, Gandini M, Freitas LP, Siqueira MM, Marinho CF, Setúbal S (2012). Profile of circulating levels of IL-1Ra, CXCL10/IP-10,
CCL4/MIP-1β and CCL2/MCP-1 in dengue fever and parvovirosis. Mem Inst Oswaldo Cruz.

[23] Ferreira RA, de Oliveira SA, Gandini M, Ferreira Lda C, Correa G, Abiraude FM, Reid MM, Cruz OG, Kubelka CF (2015). Circulating cytokines and chemokines associated with plasma
leakage and hepatic dysfunction in Brazilian children with dengue
fever. Acta Trop.

[24] Wong KL, Chen W, Balakrishnan T, Toh YX, Fink K, Wong SC (2012). Susceptibility and response of human blood monocyte subsets to
primary dengue virus infection. PloS one.

[25] Avirutnan P, Malasit P, Seliger B, Bhakdi S, Husmann M (1998). Dengue virus infection of human endothelial cells leads to
chemokine production, complement activation, and apoptosis. J Immunol.

[26] Juffrie M van Der Meer GM, Hack CE Haasnoot K, Sutaryo Veerman AJ (2000). Inflammatory mediators in dengue virus infection in children:
interleukin-8 and its relationship to neutrophil
degranulation. Infection and Immunity.

[27] Huang YH, Lei HY, Liu HS, Lin YS, Liu CC, Yeh TM (2000). Dengue virus infects human endothelial cells and induces IL-6 and
IL-8 production. Am J Trop Med Hyg.

[28] Talavera D, Castillo AM, Dominguez MC, Gutierrez AE, Meza I (2004). IL8 release, tight junction and cytoskeleton dynamic
reorganization conducive to permeability increase are induced by dengue
virus infection of microvascular endothelial monolayers. J. Gen. Virol.

[29] Barbosa-Lima G, Hottz ED, de Assis EF, Liechocki S, Souza TML, Zimmerman GA, Bozza FA, Bozza PT (2020). Dengue virus-activated platelets modulate monocyte
immunometabolic response through lipid droplet biogenesis and cytokine
signaling. J Leukoc Biol.

[30] Soo KM, Tham CL, Khalid B, Basir R, Chee HY (2019). IL-8 as a potential in-vitro severity biomarker for dengue
disease. Trop Biomed.

[31] WHO (1997). Dengue haemorrhagic fever: diagnosis, treatment, prevention and
control. https://apps.who.int/iris/handle/10665/41988.

[32] Macedo GA, Gonin ML, Pone SM, Cruz OG, Nobre FF, Brasil P (2014). Sensitivity and specificity of the World Health Organization
dengue classification schemes for severe dengue assessment in children in
Rio de Janeiro. PLoS One.

[33] Avirutnan P, Punyadee N, Noisakran S, Komoltri C, Thiemmeca S, Auethavornanan K (2006). Vascular leakage in severe dengue virus infections: a potential
role for the nonstructural viral protein NS1 and complement. J. Infect. Dis.

[34] Libraty DH, Young PR, Pickering D, Endy TP, Kalayanarooj S, Green S, Vaughn DW, Nisalak A, Ennis FA, Rothman AL (2002). High circulating levels of the dengue virus nonstructural protein
NS1 early in dengue illness correlate with the development of dengue
hemorrhagic fever. J Infect Dis.

[35] Reyes-Sandoval A, Ludert JE (2019). The Dual Role of the Antibody Response Against the Flavivirus
Non-structural Protein 1 (NS1) in Protection and
Immuno-Pathogenesis. Frontiers in immunology.

[36] Lin CF, Lei HY, Shiau AL, Liu HS, Yeh TM, Chen SH (2002). Endothelial cell apoptosis induced by antibodies against dengue
virus nonstructural protein 1 via production of nitric oxide. J Immunol.

[37] Chen MC, Lin CF, Lei HY, Lin SC, Liu HS, Yeh TM (2009). Deletion of the C-terminal region of dengue virus nonstructural
protein 1 (NS1) abolishes anti-NS1-mediated platelet dysfunction and
bleeding tendency. J Immunol.

[38] Cheng HJ, Lei HY, Lin CF, Luo YH, Wan SW, Liu HS (2009). Anti-dengue virus nonstructural protein 1 antibodies recognize
protein disulfide isomerase on platelets and inhibit platelet
aggregation. Molecular immunology.

[39] Wan SW, Yang YW, Chu YT, Lin CF, Chang CP, Yeh TM, Anderson R, Lin YS (2016). Anti-dengue virus nonstructural protein 1 antibodies contribute
to platelet phagocytosis by macrophages. Thromb Haemost.

[40] Patra G, Mallik S, Saha B, Mukhopadhyay S (2019). Assessment of chemokine and cytokine signatures in patients with
dengue infection: A hospital-based study in Kolkata, India. Acta Tropica.

[41] Ip PP, Liao F (2010). Resistance to dengue virus infection in mice is potentiated by
CXCL10 and is independent of CXCL10-mediated leukocyte
recruitment. J Immunol.

[42] Imad HA, Phumratanaprapin W, Phonrat B, Chotivanich K, Charunwatthana P, Muangnoicharoen S (2020). Cytokine Expression in Dengue Fever and Dengue Hemorrhagic Fever
Patients with Bleeding and Severe Hepatitis. Am J Trop Med Hyg.

[43] Nanda JD, Jung CJ, Satria RD, Jhan MK, Shen TJ, Tseng PC, Wang YT, Ho TS, Lin CF (2021). Serum IL-18 Is a Potential Biomarker for Predicting Severe Dengue
Disease Progression. J Immunol Res.

[44] Gowri Sankar S, Alwin Prem Anand A (2021). Cytokine IP-10 and GM-CSF are prognostic biomarkers for severity
in secondary dengue infection. Hum Immunol.

[45] Fagundes CT, Costa VV, Cisalpino D, Amaral FA, Souza PR, Souza RS (2011). IFN-gamma production depends on IL-12 and IL-18 combined action
and mediates host resistance to dengue virus infection in a nitric
oxide-dependent manner. PLoS Neglected Tropical Diseases.

[46] Pacsa AS, Agarwal R, Elbishbishi EA, Chaturvedi UC, Nagar R, Mustafa AS (2000). Role of interleukin-12 in patients with dengue hemorrhagic
fever. FEMS Immunology and Medical Microbiology.

[47] Nwe KM, Ngwe Tun MM, Myat TW, Sheng Ng CF, Htun MM, Lin H (2022). Acute-phase serum cytokine levels and correlation with clinical
outcomes in children andadults with primary and secondary dengue virus
infection in Myanmar between 2017 and 2019. Pathogens.

[48] Lin CF, Chiu SC, Hsiao YL, Wan SW, Lei HY, Shiau AL (2005). Expression of cytokine, chemokine, and adhesion molecules during
endothelial cell activation induced by antibodies against dengue virus
nonstructural protein 1. J Immunol.

[49] Nightingale ZD, Patkar C, Rothman AL (2008). Viral replication and paracrine effects result in distinct,
functional responses of dendritic cells following infection with dengue 2
virus. Journal of Leukocyte Biology.

[50] Ho LJ, Hung LF, Weng CY, Wu WL, Chou P, Lin YL (2005). Dengue virus type 2 antagonizes IFN-alpha but not IFN-gamma
antiviral effect via down-regulating Tyk2-STAT signaling in the human
dendritic cell. J Immunol.

[51] Rodriguez-Madoz JR, Belicha-Villanueva A, Bernal-Rubio D, Ashour J, Ayllon J, Fernandez-Sesma A (2010). Inhibition of the type I interferon response in human dendritic
cells by dengue virus infection requires a catalytically active NS2B3
complex. Journal of Virology.

[52] Starr R, Fuchsberger M, Lau LS, Uldrich AP, Goradia A, Willson TA (2009). SOCS-1 binding to tyrosine 441 of IFN-gamma receptor subunit 1
contributes to the attenuation of IFN-gamma signaling in
vivo. J Immunol.

[53] Munoz-Jordan JL (2010). Subversion of interferon by dengue virus. Curr Top Microbiol.

[54] Munoz-Jordan JL, Sanchez-Burgos GG, Laurent-Rolle M, Garcia-Sastre A (2003). Inhibition of interferon signaling by dengue
virus. Proc Natl Acad Sci U S A.

[55] Perera DR, Ranadeva ND, Sirisena K, Wijesinghe KJ (2024). Roles of NS1 Protein in Flavivirus Pathogenesis. ACS Infect Dis.

[56] Allione A, Bernabei P, Bosticardo M, Ariotti S, Forni G, Novelli F (1999). Nitric oxide suppresses human T lymphocyte proliferation through
IFN-gamma-dependent and IFN-gamma-independent induction of
apoptosis. J Immunol.

